# Silent Myocardial Infarction and Acute Multiorgan Failure in a COVID-19 Patient: A Case Report

**DOI:** 10.31729/jnma.6933

**Published:** 2021-10-31

**Authors:** Nischit Baral, Andrea Montalbano, Ashiya Khan, Mehak Qureshi, Pankaj Luitel

**Affiliations:** 1 Department of Internal Medicine, Mciaren Flint/Michigan State University College of Human Medicine, United States of America; 2 Department of Internal Medicine, Michigan State University College of Human Medicine, United States of America; 3 Department of Internal Medicine, AMITA Health/Saint Francis Hospital, United States

**Keywords:** *case reports*, *COVID-19*, *myocardial infarction*, *ST elevation myocardial infarction*

## Abstract

Silent myocardial infarction or unrecognized myocardial infarction has increased prevalence in elderly population with increased cardiovascular risk factors. However, its prevalence in COVID-19 patients is not well-known. A 77-year-old Caucasian male with COVID-19 pneumonia, presented with silent ST-segment elevation myocardial infarction, diabetic ketoacidosis and multiorgan failure. He underwent cardiac catheterization and drug eluting stent placement in the ostial right coronary artery with safety protocol. He was discharged to extended-care-facility in stable condition. This is a first case report of silent ST-segment elevation myocardial infarction in a patient presenting with COVID-19. In patients with COVID-19, acute myocardial infarction should be ruled out even when asymptomatic, especially in older patients. Prompt intervention using safety protocol is life-saving.

## INTRODUCTION

Silent myocardial infarction or unrecognized myocardial infarction has a highly variable prevalence but is more common in patients with cardiovascular risk factors, hypertension, longer duration of diabetes mellitus, and the elderly population.^1^ As per the Centers for Disease Control and Prevention (CDC), More than 33.5 million cases of COVID-19 have been diagnosed.^2^ However, little is known about its prevalence in COVID-19 patients. We presented a case of a 77-year-old Caucasian male with COVID-19 pneumonia, presented with silent ST-segment elevation myocardial infarction, diabetic ketoacidosis, and multiorgan failure. He underwent cardiac catheterization and drug-eluting stent placement in the ostial right coronary artery with safety protocol.

## CASE REPORT

Silent myocardial infarction or unrecognized myocardial infarction has highly variable prevalence but is more common in patients with cardiovascular risk factors, hypertension, longer duration of diabetes mellitus, and elderly population.^1^ However, little is known about its prevalence in COVID-19 patients. A 77-year-old Caucasian male presented to the emergency department with symptoms of shortness of breath, cough, and fever from three days prior to admission. Upon arrival, the patient was desaturating with oxygen saturation(SpO2) of 80% on 10liters nasal cannula. He was put on a non rebreather mask with an oxygen rate of 15L/min, after which oxygen saturation improved to 95%. His other vitals were: systolic blood pressure (SBP) 120130mm of Hg, heart rate 80 beats per minute. He was afebrile. Physical examination was significant for right sided crepitations.

His past medical history is notable for ischemic cardiomyopathy with ejection fraction(EF) 20-25% for which he underwent stenting of the proximal left anterior descending and proximal right coronary artery one and half years ago, insulin-dependent diabetes, and dyslipidemia. Patient's wife was recently diagnosed with COVID-19 pneumonia.

**Figure 1:**
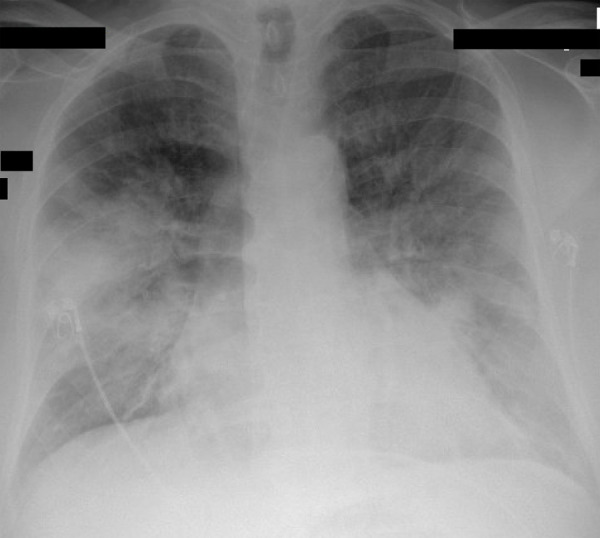
Chest X-ray at day of admission showing diffuse bilateral infiltrates greater on the right than left.

On admission, his arterial blood gas (ABG) showed pH of 7.364, pCO_2_ of 28.7mmHg, pO_2_ of 81.9mmHG, and HCO_3_^-^ of 16mmol/L, suggestive of metabolic acidosis with respiratory compensation. Comprehensive metabolic panel(CMP) was remarkable for a glucose of 541mg/dL, Creatinine of 2.66mg/dL (baseline 1.5mg/dL), potassium of 5.8mmol/L, and an anion gap of 22mmol/L. His beta-hydroxybutyrate level was high (23.1mg/dl), intact PTH high (102.3pg/ml), and initial lactic acid was elevated to 5.1mM/L. Complete blood count(CBC) was within normal limits but within 48 hours his white blood cell(WBC) count increased from 10.33*10^3^/uL to 21.40 x 10^3^/uL. His chest X-ray showed diffuse bilateral infiltrates greater on the right than left (Figure 1).

**Figure 2:**
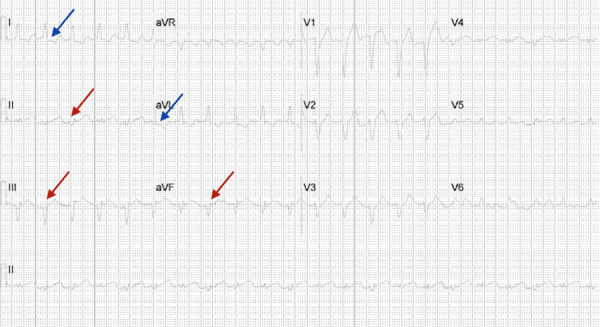
EKG showing 1 mm ST segment elevation in lead II and aVF and 2 mm ST segment elevation in lead III (red arrows) with ST segment depressions in Leads I and aVL (blue arrows), suggestive of an acute inferior wall myocardial infarction.

His SARS-Cov-2 RNA rapid rest was positive on admission. Serum inflammatory markers were all elevated on admission including serum lactate dehydrogenase (LDH) 252U/L, C-Reactive Protein (CRP) 6.4mg/dl, Ferritin 351.3ng/ml and D-dimer 0.79 mg/L fibrinogen equivalent unit. His initial troponin- I high sensitivity was 0.923ng/ml, which subsequently increased to 21.422ng/ml and peaked at 23.684ng/ml over 6 hours. Electrocardiogram(EKG) showed 1-2 mm ST segment elevation in lead II, III, aVF with ST segment depressions in Leads I and aVL, suggestive of an acute inferior wall myocardial infarction (Figure 2).

His Brain Natriuretic Peptide (BNP) was elevated to 1888pg/ml and initial echocardiography showed severely decreased left ventricular systolic function (EF15-20%) with global left ventricular hypokinesis.

**Figure 3:**
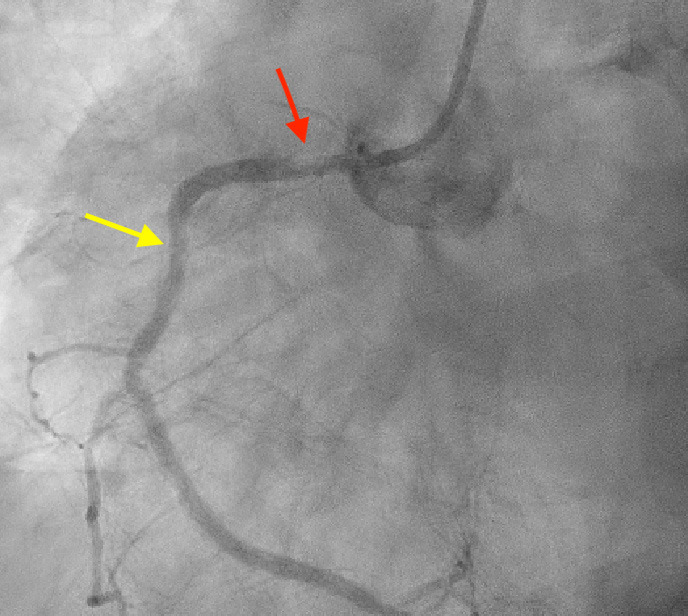
Left heart catheterization revealed a 95% occlusion of the RCA at the ostium (red arrow) and mild haziness in the previously deployed stent (yellow arrow) in proximal RCA.

In the ED, his home medications of Aspirin, Clopidogrel, Atorvastatin and Metoprolol were resumed. IV heparin was started for anticoagulation (low molecular weight heparin was not given in view of AKI) for elevated troponins. IV insulin was started as per DKA protocol. Patient was taken immediately for left heart catheterization (LHC) following adequate COVID-19 safety protocols and personal protective equipment (PPE). Left heart catheterization revealed a 95% occlusion of the Right Coronary Artery (RCA) at the ostium, hazy filling defect in the previously deployed stent in the proximal RCA (Figure 3).

**Figure 4:**
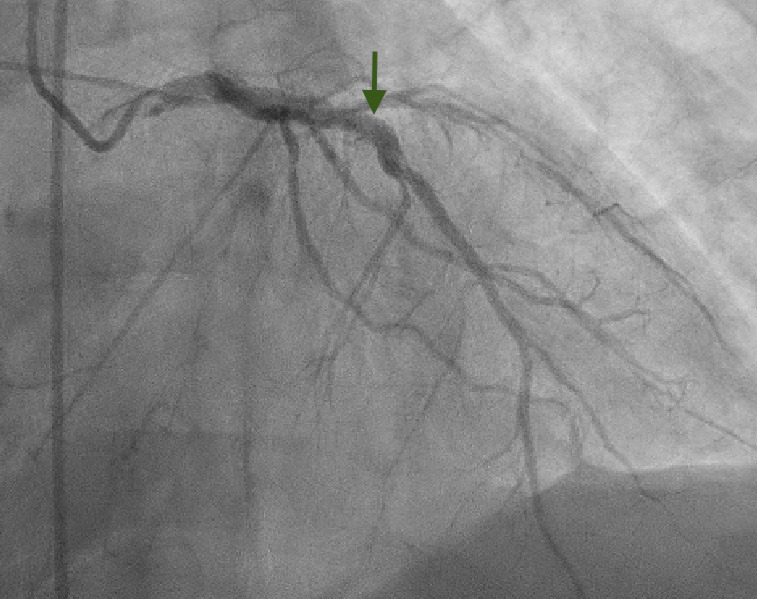
Left heart/catheterization revealed a patent stent (arrow) in the LAD.

In addition to this, the left heart catheterization showed a widely patent stent in proximal left anterior descending (LAD) (Figure 4).

**Figure 5:**
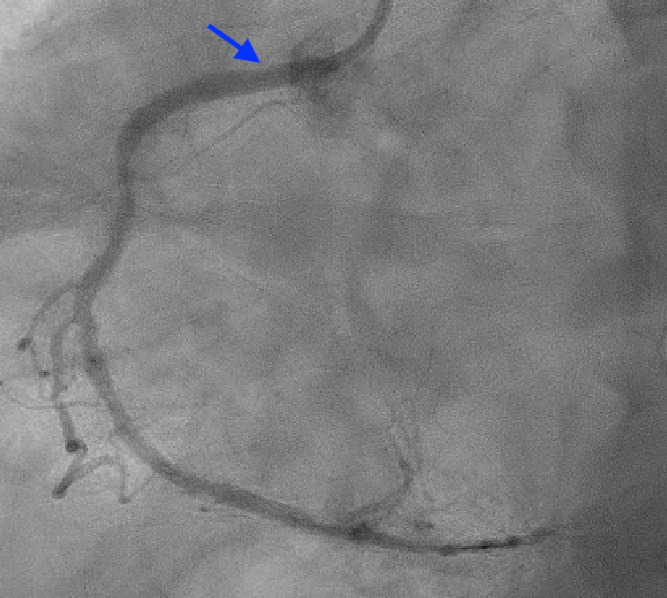
Left heart catheterization after drug eluting stent placement to proximal RCA with no residual stenosis (arrow).

He underwent drug eluting stent placement to ostial RCA with no residual stenosis. Thrombolysis in myocardial infarction (TIMI) flow grade 3 was present post-procedure (Figure 5).

After the catherization, his EKG changes were resolved. In the Intensive Care Unit (ICU), he was started on Intravenous (IV) ceftriaxone, azithromycin, dexamethasone, and breathing treatments for COVID-19 pneumonia. After four days of admission, his kidney function further declined and he required temporary hemodialysis. After 6 days, he had an episode of coffee ground emesis, with hemoglobin dropping from 12.8g/dL in admission to 7.1g/dL. He was transfused with packed red blood cells (RBC). IV pantoprazole drip was started with close monitoring of hemoglobin. There were no subsequent episodes of hematemesis and his hemoglobin improved to 8.4g/dL. After 10 days, he started on enoxaparin at 40mg twice daily due to elevated D-dimer. However, after 11 days, the patient developed swelling in his left lower extremity and venous doppler showed acute deep vein thrombosis (DVT). His anticoagulation was changed to Eliquis 2.5 twice daily, to be taken for 3 months. After 20 days, the patient's kidney function improved and he was monitored off dialysis. His repeat Echocardiogram improved EF of 25-30%. After 32 days, the patient's functional capacity improved and he was discharged to an extended care facility with instructions for continued follow up.

## DISCUSSION

As per Centers for Disease Control and Prevention (CDC), More than 33.5 million cases of COVID-19 have been diagnosed and the death rate is estimated to be 180 per 100,000 in the United States (US) alone.^2^ We report here on a case of a man with no chest pain or typical angina who initially presented with symptoms of COVID-19 but was discovered to have a STEMI, Diabetic Ketoacidosis (DKA), Acute Kidney Injury (AKI), and multiorgan dysfunction. The unique qualities we wish to explicate is the possibility of a silent MI, DKA, and multiorgan dysfunction secondary to infection with COVID-19. We would like to convey the importance of early recognition of silent acute myocardial infarction (AMI) in these patients. Endothelial injury by complement activation, hyperviscosity, and increased procoagulant factors during COVID-19 is not uncommon. ^3,4^ COVID-19 causes hyperinflammatory state, which ultimately results in multiorgan dysfunction and increase in morbidity and mortality.^5^ Moreover, novel coronavirus binds with the angiotensin-converting enzyme-2 (ACE-2) through which it can infect and spread rapidly throughout the body.^6^

Our study is unique because we report a first case of silent ST-segment myocardial infarction (STEMI) as an initial clinical manifestation of COVID-19. There was no intracardiac stent restenosis as the lesion was on a new site. Our patient also had DKA, however, he was not having diabetic neuropathy as a complication of diabetes, which could explain the silent AMI. Silent AMI was reported during convalescence phase of COVID-19 infection in a case report by Tschope et al unlike in our patient where silent AMI was reported during COVID-19 infection.^7^ There is lack of enough evidence on the exact mechanism of silent AMI in COVID patients. Our patient had a patient stent in his LAD which ruled out intracoronary stent restenosis (ISR). There is also lack of sufficient data on ISR in COVID-19 patients.^8^ In COVID-19, multiple complications within the cardiovascular system can arise, including myocardial injury, myocarditis, arrhythmias, or heart failure, which can further complicate the clinical picture .^9^ Our case report conveys the importance of safety protocols and PPE in catheterization lab in times of pandemic. With proper safety protocols, patients with silent STEMI can get successful intervention. Prompt recognition and aggressive management of silent STEMI is life-saving.

The patient is scheduled to follow-up in cardiology and pulmonary clinic, but no follow up has been conducted to date. His chest radiograph at day 14 and day 30 after admission showed improvement in the bilateral opacities. Silent STEMI can be a unique presentation of COVID-19. Prompt diagnosis and intervention following safety protocols can be life-saving.

## References

[1] Valensi P, Lorgis L, Cottin Y (2011). Prevalence, incidence, predictive factors and prognosis of silent myocardial infarction: a review of the literature.. Arch Cardiovasc Dis..

[2] CDC Case Surveillance Task Force. (2021). COVID-19 Case Surveillance Public Use Data [Internet].

[3] Ranucci M, Ballotta A, Di Dedda U, Bayshnikova E, Dei Poli M, Resta M (2020). The procoagulant pattern of patients with COVID-19 acute respiratory distress syndrome.. J Thromb Haemost..

[4] Ma L, Sahu SK, Cano M, Kuppuswamy V, Bajwa J, McPhatter J (2021). Increased complement activation is a distinctive feature of severe SARS-CoV-2 infection.. Sci Immunol..

[5] Mehta P, McAuley DF, Brown M, Sanchez E, Tattersall RS, Manson JJ (2020). COVID-19: consider cytokine storm syndromes and immunosuppression.. Lancet..

[6] Hamming I, Timens W, Bulthuis ML, Lely AT, Navis G, van Goor H (2004). Tissue distribution of ACE2 protein, the functional receptor for SARS coronavirus. A first step in understanding SARS pathogenesis.. J Pathol..

[7] Tschöpe C, Sherif M, Anker MS, Geisel D, Kuehne T, Kelle S (2021). COVID-19-convalescence phase unmasks a silent myocardial infarction due to coronary plaque rupture.. ESC Heart Fail..

[8] Hamadeh A, Aldujeli A, Briedis K, Tecson KM, Sanz-Sanchez J, Al Dujeili M (2020). Characteristics and Outcomes in Patients Presenting With COVID-19 and ST-Segment Elevation Myocardial Infarction.. Am J Cardiol..

[9] Long B, Brady WJ, Koyfman A, Gottlieb M (2020). Cardiovascular complications in COVID-19.. Am J Emerg Med..

